# Mammograms in 35-year-old to 39-year-old symptomatic patients

**DOI:** 10.1186/bcr3320

**Published:** 2012-11-09

**Authors:** KA Lim, P Young

**Affiliations:** 1 Cardiff and Vale University Health Board, Cardiff, UK

## Introduction

Currently, in our institution, all women presenting at the Triple Assessment Symptomatic Breast Clinic over 35 years of age undergo two-view mammograms. Recent recommendations suggest that mammograms should only be performed above the age of 40 [1]. We reviewed our patients to see whether changing our policy would have resulted in any missed cancers.

## Methods

All patients on our Breast Cancer Database diagnosed between April 2002 and March 2012, aged between 35 and 39 years at diagnosis, were identified. Patient records were reviewed to determine how the cancer was diagnosed and treated.

## Results

Fifty-one patients were identified; 11 were diagnosed elsewhere, and therefore excluded, and one was screen-detected and also excluded. Of the remaining 39 patients, one patient was male. In 33/39 (84.6%) patients, ultrasound was performed of a palpable lesion and mammography did not add any additional diagnostic value. Of the remaining six patients, three had obvious clinical signs of malignancy. The remaining three patients had malignancy diagnosed as the result of their mammograms. Two (5.1%) had mammographic microcalcification due to intermediate-grade DCIS and one patient had an impalpable cancer, seen on the mammogram, which prompted the diagnostic ultrasound.

## Conclusion

In our institution, over a 10-year period, two cases of intermediate-grade DCIS and one case of Grade 2 invasive ductal carcinoma would have been missed if routine mammography had not been performed in the 35 to 39 year age group.

## References

[B1] Willett AM, Michell MJ, Lee MJR Best Practice Diagnostic Guidelines for Patients Presenting with Breast Symptoms 2010 London: Department of Health

